# Continuous contractile force and electrical signal recordings of 3D cardiac tissue utilizing conductive hydrogel pillars on a chip

**DOI:** 10.1016/j.mtbio.2023.100626

**Published:** 2023-04-06

**Authors:** Feng Zhang, Hongyi Cheng, Kaiyun Qu, Xuetian Qian, Yongping Lin, Yike Zhang, Sichong Qian, Ningping Huang, Chang Cui, Minglong Chen

**Affiliations:** a Department of Cardiology, The First Affiliated Hospital of Nanjing Medical University, Nanjing, Jiangsu, 210000, China; b State Key Laboratory of Bioelectronics, School of Biological Science and Medical Engineering, Southeast University, Nanjing, 210096, China; c Gusu School, Nanjing Medical University, The Affiliated Suzhou Hospital of Nanjing Medical University, Suzhou, Jiangsu, 215002, China; d Department of Gastroenterology, Nanjing First Hospital, Nanjing Medical University, No. 68 Changle Road, Nanjing, 210006, China; e Department of Cardiac Surgery, Beijing Anzhen Hospital, Beijing, 100029, China; f Key Laboratory of Targeted Intervention of Cardiovascular Disease, Collaborative Innovation Center for Cardiovascular Disease Translational Medicine, Nanjing Medical University, Nanjing, Jiangsu, 210000, China

**Keywords:** Heart-on-chip, Cardiac tissue engineering, *In situ* monitoring, Hydrogel pillar electrodes, Electrical stimulation

## Abstract

Heart-on-chip emerged as a potential tool for cardiac tissue engineering, recapitulating key physiological cues in cardiac pathophysiology. Controlled electrical stimulation and the ability to provide directly analyzed functional readouts are essential to evaluate the physiology of cardiac tissues in the heart-on-chip platforms. In this scenario, a novel heart-on-chip platform integrating two soft conductive hydrogel pillar electrodes was presented here. Human-induced pluripotent stem cell-derived cardiomyocytes (hiPSC-CMs) and cardiac fibroblasts were seeded into the apparatus to create 3D human cardiac tissues. The application of electrical stimulation improved functional performance by altering the dynamics of tissue structure and contractile development. The contractile forces that cardiac tissues contract was accurately measured through optical tracking of hydrogel pillar displacement. Furthermore, the conductive properties of hydrogel pillars allowed direct and non-invasive electrophysiology studies, enabling continuous monitoring of signal changes in real-time while dynamically administering drugs to the cardiac tissues, as shown by a chronotropic reaction to isoprenaline and verapamil. Overall, the platform for acquiring contractile force and electrophysiological signals *in situ* allowed monitoring the tissue development trend without interrupting the culture process and could have diverse applications in preclinical drug testing, disease modeling, and therapeutic discovery.

## Highlights

• A heart-on-chip platform for 3D cardiac tissues generation.
• Novel soft conductive pillar electrodes integration with stimulation capabilities.
• Reproducible contractile quantification of 3D cardiac tissues.
• High-quality electrophysiology measurement in *real-time* during drug testing.

## Graphical abstract

**Figure:**
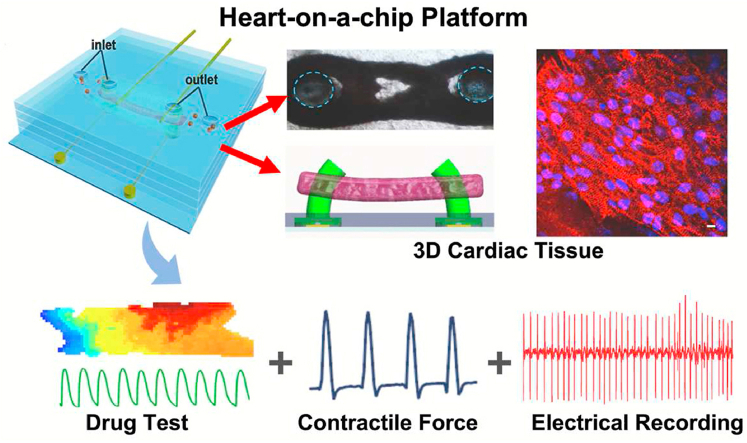

## 1 Introduction

The main cause of death worldwide is cardiovascular disease (CVD) [1]. Meanwhile, drug development is an extremely costly endeavor. A new medicine will take an average of 10–15 years and more than US$2 billion before it can reach the pharmacy shelf [2]. The high attrition rate has hampered drug development for CVD due to cardiac toxicity in multiple failed late-phase clinical trials and drug withdrawal during post-marketing surveillance, which indicates that current animal and *in vitro* cell culture models do not adequately predict cardiovascular side effects [3]. Therefore, the development of relevant human heart models represents an urgent need to improve the prediction of cardiotoxicity in the early preclinical stage.

The growing technology of heart-on-chip paired with cardiac cells enables a viable alternative approach [[4], [5], [6]]. The heart-on-chip technologies are intended to faithfully reproduce the complicated native cardiac tissue by supplying biochemical and biophysical signals [7,8]. Heart-on-chip technology has advanced due to the development of regenerative medicine by incorporating human-induced stem cell-derived cardiomyocytes (hiPSC-CMs) into the device [9]. Many heart-on-chip systems have been found to more accurately depict several features of human heart responses than two-dimensional (2D) cell culture, making them intriguing tools for modeling cardiac disease and evaluating cardiac drugs [10,11]. However, the immature characteristics of hiPSC-CMs can only provide a preliminary investigation or understanding of human pathophysiology [12]. For more advanced insights, a chip system model integrated with external stimulation, electrophysiology, and contractility readouts is highly desired, which would dramatically increase the potential of heart-on-chip in predicting cardiac toxicity, finally reducing the associated costs in the pharmaceutical industry [[13], [14], [15], [16], [17]].

It is customary to allow cardiac cells to self-assemble into myocardial strands in the presence of an extracellular matrix environment in order to generate three-dimensional (3D) cardiac tissues on a chip [18]. Recent studies have demonstrated the fundamental role of electrical, mechanical, chemical, or combined stimulations in promoting the maturation of cardiac tissues *in vitro* [[19], [20], [21], [22]]. Due to the unique excitability of cardiomyocytes, electric stimulation is an effective method for regulating the sarcomeres’ organization, enhancing the expression of gap junctions, and improving the calcium handling process [6]. Current heart-on-chip systems integrate with electrical stimulation capability by utilizing thin-film electrodes, platinum wires, or carbon rods have been presented [[23], [24], [25]].

On the other hand, a number of strategies have successfully been implemented in heart-on-chip platforms to offer electrophysiological monitoring and proper mechanical characterization [[26], [27], [28]]. Using microfabrication techniques, multi-electrode arrays (MEA) have been used for mapping electrophysiological activity [29]. However, the majority of MEA systems now in use rely on cell monolayers to detect local action potentials with high spatial resolution but are limited in measurements in 3D models that represent the complexity of native human cardiac tissues [30]. In addition, these 2D monolayer systems can only analyze contractile features in a very restricted manner. Instead, contractility assay of 3D cardiac tissue based on elastomeric pillars or wires like polydimethylsiloxane (PDMS) is frequently used [31,32]. These flexible structures constrain and guide the cardiac cells and matrix gel into specific tissue constructs, and accurately calculate the tissue contractile force based on its deformation [33]. Nevertheless, they also come with some shortcomings. For example, due to the non-conductive properties of PDMS, there is little or no data regarding the electrophysiological measurement of 3D cardiac tissues in previous studies describing heart-on-chip platforms [34]. Although it has been reported that doping conductive materials such as Pt powder can improve the conductivity of PDMS and be used to measure the electrophysiological signal of myocardial tissue, it is limited in contractile force measurement because of the increased elastic modulus [35]. Generally, cardiac electrophysiology and contractile force are highly correlated but not identical [36]. A platform that can measure both characteristics would provide a more comprehensive assessment of tissue function. In recent years, conductive hydrogels have been developed and used in the biosensing field because of their distinct properties, such as electrical conductivity and tissue friendly mechanical properties [37]. These extraordinary properties enable conductive hydrogel-based sensors to demonstrate high performance for recognizing stimuli and detecting movement [38]. However, the application of conducting hydrogels to the detection of electrophysiological and mechanical signals of engineered tissues *in vitro* is rarely reported.

Herein, we present a novel heart-on-chip platform that developed 3D cardiac tissue and was capable of applying electrical stimulation to improve functional performance. Specifically, we designed two soft conductive hydrogel pillar electrodes integrated into the device for continuous and non-invasive monitoring of tissue signals *in situ*. The versatile platform was successfully employed to record the 3D cardiac tissue field potential with an excellent signal, enabling remote assessment of the cardiac tissue during dynamic drug delivery. Additionally, optical tracking of hydrogel pillar displacement allowed for the precise measurement of the contractile forces generated by cardiac tissues. We also showed how different electrical stimulation inside the platform affected the medication reactions in cardiac tissues.

## 2 Materials and methods

### 2.1 Device design and fabrication

The heart-on-chip device primarily consisted of a rectangular chamber (4.5 ​mm ​× ​2 ​mm ​× ​1 ​mm, L ​× ​W ​× ​H), interconnected by two 0.7 ​mm diameter parallel platinum (Pt) wires. Inside the chamber were two hydrogel pillar electrodes (1 ​mm ​× ​0.5 ​mm, H ​× ​D) connected to gold (Au) electrodes on the bottom glass substrate. Utilizing CAD software (AutoCAD, Autodesk Ink), photomasks for chip production were developed and printed at high resolution. Standard photolithography methods were used to create the SU-8 Master molds from the photomasks [39]. By combining PDMS base and curing agent at a 10:1 mass ratio, devices were manufactured out of PDMS. The Au electrodes on the bottom glass substrate were prepared using standard vacuum sputtering coating techniques. First, using a “lift-off” technique, Au interconnects were micropatterned on a glass substrate (200 ​nm Au and 20 ​nm Ti beneath). Si3N4 was used to avoid scratching the Au electrode in the passivation layer. Second, we modified the Au surface with polydopamine to improve hydrogel-Au adhesion according to a previous report [40]. The conductive hydrogels were prepared by a simple protocol. Briefly, the cross-linker genipin was dissolved (Zhixin Biotechnology, China) in water, yielding 0.3% (w/v) solution. The gelatin-PEDOT: PSS solution was prepared by dissolving 6% (w/v) of gelatin powder (10−70 ​kDa, extracted from bovine skin, Shengxing Biotechnology, China) in 0.1, 0.2 or 0.3 ​wt % PEDOT: PSS solution (Sigma, 739324, diluted with ultrapure water), and gelling was allowed to proceed for 1 ​h at 4 ​°C. Finally, the hydrogels were further crosslinked in genipin solution at room temperature for 24 ​h. To fabricate the pillar electrodes, a poly(methyl methacrylate) (PMMA) plate mold with micro-holes was used. Briefly, A layer of PDMS was attached between the glass substrate and PMMA plate, and the Au electrodes were exposed. Then, 5 ​μL of gelatin-PEDOT:PSS solution was dropped on the micro-holes of the PMMA plate, then slowly vacuumed at 37 ​°C to fill the holes with the solution. After cross-linking, remove the PMMA plate and excess hydrogel on the surface. In order to prevent the hydrogel pillar from slipping off, the bottom layer was initially treated with dopamine solution for 30 ​min to generate a polydopamine film, which improved the adhesion between the hydrogel pillar and the bottom layer. Also, to prevent loose connections, the design of the snap structure ensures that the pillar is firmly fastened at the base of the chamber after processing (Fig. 2a iii-v). The device was sterilized before cell seeding by rinsing with 75% ethanol and then three washes with phosphate-buffered saline (PBS).

### 2.2 Hydrogel pillar electrodes characterization

Using a BioScope Resolve atomic force microscope (AFM, Bruker Corporation) with a cantilever (PFQNM-LC Probe, k ​= ​0.133 ​N/m) at 0.5 ​Hz scan rate and 2 ​kHz peak force frequency, the Young's modulus of hydrogel pillar electrodes in PBS was evaluated. The structure morphology of the hydrogel pillar was observed using a scanning electron microscope (SEM, Ultra Plus, Carl Zeiss). After being frozen in liquid nitrogen for 48 ​h, SEM samples were freeze-dried. Rheological measurements were performed using an Anton-Paar MCR302 rheometer using parallel plates at 37 ​°C in oscillatory mode. Briefly, the hydrogel sample was applied to the rheometer and the plate of the rheometer was then moved down, and brought in contact with the hydrogel. The storage modulus (G′) and loss modulus (G″) were recorded as functions of frequency (1–100 ​rad/s). The creep recovery of the hydrogel was measured over time.

For impedance analysis, while sandwiched between two indium tin oxides (ITO), cylindrical hydrogels were scanned for frequencies between 0.1 ​Hz and 1 ​MHz using a CHI600E electrochemical workstation. The capacitance retention behaviors of the hydrogels were calculated using a CV test (voltage range of −1 to 1 ​V, scan rate of 100 ​mV ​s^−1^). The electrical conductivity of flacks (diameter 20 ​mm, height 3 ​mm) of hydrogels was measured using the standard Van Der Pauw four-point probe method (ST2242, China). The Cell Counting Kit-8 (CCK-8, Dojindo) assay was used to gauge the biocompatibility of hydrogels. Cardiomyocytes were cultured on the hydrogel at a cell density of 20 ​000/cm^2^. The cells were incubated with the CCK-8 and cell culture media at a volume ratio of 1:10 for 2 ​h after being cultivated for 1, 3, and 5 days. A microplate reader (Biotek Epoch) was used to determine the mixture's absorbance at 450 ​nm.

### 2.3 Cardiac tissue construction

Human-induced pluripotent stem cells (hiPSCs) were used to create ventricular cardiomyocytes through a PSC Cardiomyocyte Differentiation Kit (ThermoFisher Scientific A2921201). From day 7 following differentiation induction, contracting cells were seen, and cell purification (Cellapy CA2005100) was performed to obtain high-purity cardiomyocytes. Collagen type Ⅰ from rat tail (Corning 354249) and Geltrex (LDEV-Free, Gibco A1413202) were used to fabricate cardiac tissues around the hydrogel pillars. The final concentrations were collagen I (3 ​mg/mL), Geltrex (0.08 ​mg/mL), 1% RevitaCell supplement (Gibco A4238401), 2.3% 1 ​N NaOH, and 3 ​× ​10^7^ total cells/mL composed of 10% human cardiac fibroblasts (cFBs) (Procell C-12375) and 90% cardiomyocytes [24]. A volume of 15 ​μl of this cocktail was seeded into the cell loading chamber through the inlet port of the chip device. After 15 ​min gelation at 37 ​°C, devices were kept in a typical cell culture incubator while RPMI with B27 supplement (Gibco 17504-044) medium was injected into the side channels at a flow rate of 20 ​μL/min through the lateral inlet port.

### 2.4 Electrical pacing of cardiac tissues

Using a cardiac stimulator (MODEL 4100, A-M Systems) and Pt wires, electrical pacing of 3D cardiac tissues was performed. The application of field pacing was initiated after one day of cell preculture. Biphasic electrical pacing (2 ​ms duration, 3–4 ​V/cm) was carried out at 2 ​Hz, and then the frequency was increased by 1 ​Hz per day until it reached 6 ​Hz. Cardiac tissues were then maintained at 1 ​Hz until day 7.

### 2.5 Immunofluorescence staining

At room temperature, cardiac tissues were fixed inside the chips with 4% paraformaldehyde for 1 ​h. For immunostaining, tissues were embedded at optimal cutting temperature and cryo-sectioned to 20 ​μm thickness. After permeabilization (0.2% Triton X-100), 3% bovine serum albumin (BSA) was used to block the samples. The following primary antibodies were used for immunostaining: mouse *anti*-α-actinin (Sarcomeric) (Sigma-Aldrich SAB4200813; 1:200); mouse *anti*-cTnI (Sigma-Aldrich MFCD01864286; 1:200); rabbit *anti*-connexin-43 (Cell Signaling 3512 ​S; 1:75); and the proper secondary antibodies: Alexa Fluor 594 goat anti-mouse IgG (Invitrogen A-21044, 1:200); Alexa Fluor 488 goat anti-mouse IgG (Invitrogen A-10680, 1:200); Alexa Fluor 594 goat anti-rabbit IgG (Invitrogen A-11037, 1:200). The nucleus was stained with DAPI (Beyotime, 1:200). A Revolution XD confocal laser scanning microscope (Andor) was used for imaging.

### 2.6 Measurement of contraction force

With custom-built motion tracking software, bright-field movies were examined for contraction force measurement. Briefly, the video analysis is based on the Horn-Schunck optical flow algorithm, which precisely calculates the differences between adjacent frames by comparing the correlation between adjacent frames and the changes of pixels of the image sequence in the time domain [41]. Based on the calculated optical flow matrices, the pillars' original coordinates at frame 0 of each recording were obtained, finally quantified as absolute deflection motion during contractions [42]. The lateral deflection of the hydrogel pillars is connected to contractile force based on the idea of beam-bending theory by F = (3πED^4^/64 ​L^3^)δ (F, E, D, L, and δ are the exerted force, Young's modulus, diameter, height, and deflection of the pillar, respectively). It is important to note that the deflection is the actual pulling displacement, which is not affected by passive tension. All videos collected in our experiment were shot at a fixed frame rate of 25 frames/s under the same light intensity.

### 2.7 Calcium transient measurement

Heart-on-chip devices were maintained in a constant temperature environmental chamber at 37 ​°C, and the chamber was subsequently filled with 5 ​μM calcium indicator Fluo-4 AM dye for 30 ​min. The calcium testing procedure was carried out with the tissue beating spontaneously and with a 1 ​Hz stimulation. Calcium transient videos were recorded with a high frame speed complementary metal-oxide-semiconductor (CMOS) camera (Tucsen, Dhyana95) and processed in ImageJ software. To figure out the relative variations in intracellular calcium, dF/F0 was determined. The rate of change in calcium transient fluorescence was examined using a MATLAB script.

### 2.8 Field potential recording and signal analysis

The stimulated and control groups were kept at 37 ​°C, field potential of the developed human cardiac tissues was recorded during spontaneous beating through the conductive hydrogel pillars and the bottom gold electrodes. Field potential signals were acquired with a rate of 250 samples/s using a biosensing platform (OpenBCI Cyton Biosensing Board) connected to a personal computer. For drug testing, 1 ​μM isoprenaline in RPMI with B27 medium was infused into the cardiac tissues. The tissues were incubated for 5 ​min, followed by a 2-min recording of field potential. The beating rate variability was analyzed using Kubios HRV software (Version 3.4.0, Kuopio).

### 2.9 Statistics

Prism 8 was utilized for the statistical analysis. Student's t-test was used to compare the data between two groups. Statistical significance was accepted at the *p* ​< ​0.05 level and indicated in figures as ∗*p* ​< ​0.05, ∗∗*p* ​< ​0.01, ∗∗∗*p* ​< ​0.001.

## 3 Results and discussion

### 3.1 Heart-on-chip design

**Fig. 1:**
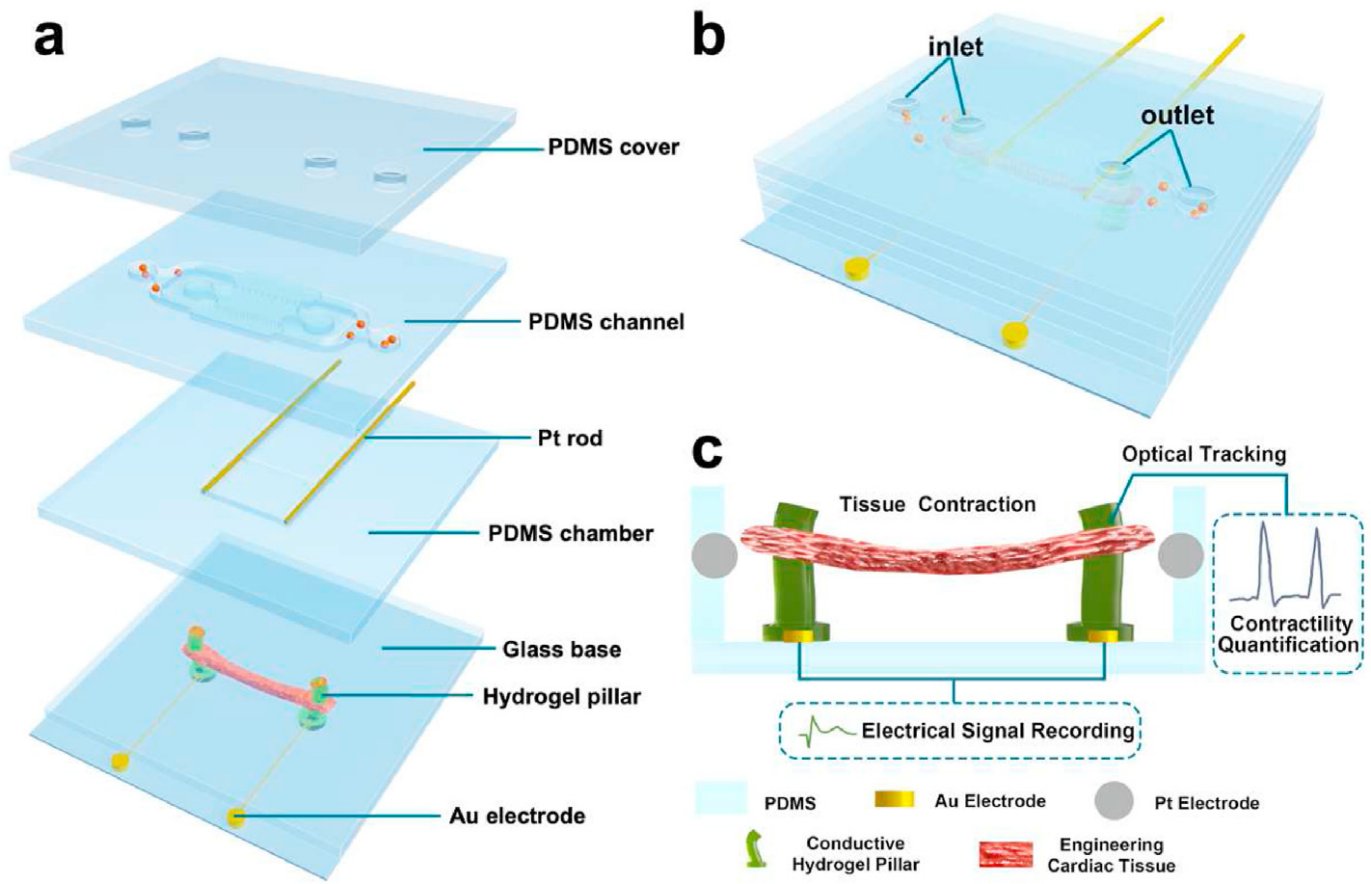
Heart-on-chip setup and characteristics. (a) Schematic diagram of the chip device, including inlet/outlet ports, microchannel, PDMS culture chamber, Pt rods, and glass base integrated with Au and conductive hydrogel pillar electrodes. (b) 3D schematic of the heart-on-a-chip device. (c) Schematic side view of the cardiac tissue, hydrogel pillar electrodes, and culture chambers (not to scale).

To evaluate the electrical activity and contractile force of 3D beating cardiac tissues induced by electrical stimulation, the heart-on-chip device was modified from our prior platform [24]. Fig. 1a shows a sketch of a representative device composed of four layers, each 1 ​mm in thickness. All layers were stacked and packaged to form a complete perfusable device (Fig. 1b). The rectangle shape of the culture chamber integrated with two conductive hydrogel pillars was designed for the fabrication of the cardiac tissues' uniaxial alignment. This configuration serves as a template for cardiac cytoskeletal remolding to replicate the structure of the heart tissue *in vivo*, as previously described [43]. A schematic side view of the culture chamber was presented in Fig. 1c, highlighting the integrated Au and hydrogel pillar electrodes. The seeded cardiomyocytes and human cardiac fibroblasts formed a 3D cardiac tissue anchored mechanically around the pillar electrodes. This columnar electrode design takes electrophysiology into consideration that can be used to record the local field potential of the cardiac tissues in real-time. Tissue contraction-induced periodic displacements of pillar electrodes were used for contractile force quantification.

**Fig. 2:**
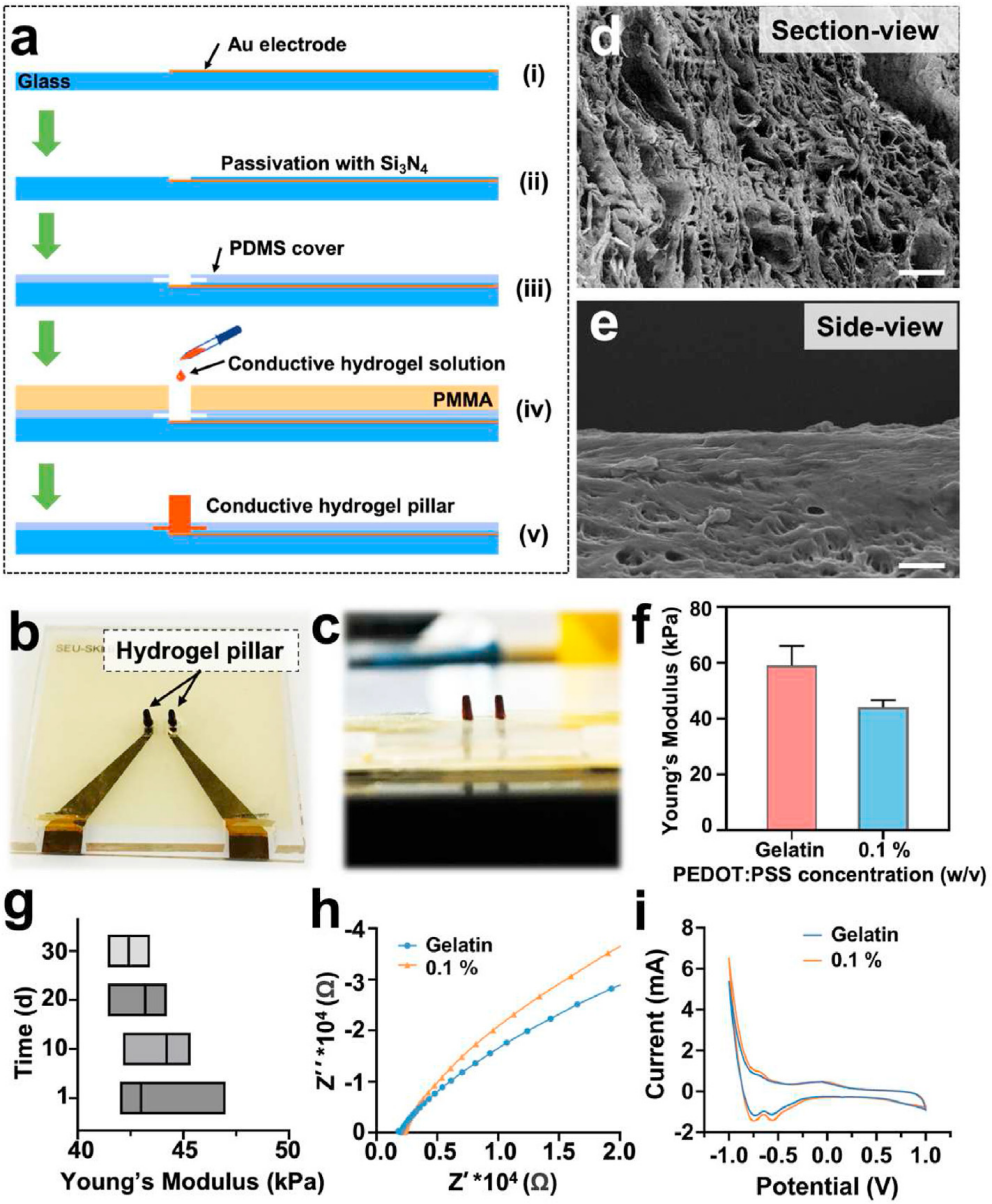
Fabrication and characterization of the hydrogel electrodes. (a) General overview of the fabrication process of hydrogel pillar electrodes. (b, c) Photos of the top-view and side-view of conductive hydrogel pillar electrodes. (d) SEM images of pillar electrode with section-view and e) side-view of the electrodes. Scale bar: 50 ​μm. (f) AFM measurement of Young's modulus of PEDOT: PSS-incorporated gelatin-based hydrogels. (g) The Young's Modulus of PEDOT: PSS-incorporated gelatin-based hydrogels was comparable during 1 month of culture with cells and media (n ​≥ ​3). (h) Nyquist plots of different hydrogels in PBS. (i) Cyclic voltammetry plot of different hydrogels over a range from −1 to 1 ​V at a scan rate of 100 ​mV ​s^−1^.

### 3.2 Fabrication of the hydrogel electrodes

Recent studies have demonstrated that 3D electrodes, e.g., Pt-PDMS pillar electrodes and pyrolytic carbon micropillars electrodes, are able to achieve field potential recording [35,44]. However, compared to cardiac tissues (10–100 ​kPa), these materials' Young's modulus is several orders of magnitude larger, leading to a significant mechanical mismatch affecting tissue contractility [45]. Previous studies have shown that cardiomyocytes generate greater mechanical force on substrates with higher stiffness (4.4–99.7 ​kPa) and the greatest cell area on a substrate stiffness of 49.4 ​kPa [46]. Therefore, besides electrochemical properties, the mechanical properties of the electrodes should be taken into consideration. We hypothesized that conductive hydrogels were ideal materials for mechanically matched electrodes because of their tissue-like softness [38,47]. In this work, we used gelatin-based hydrogel to fabricate conductive pillar electrodes based on our previous study [48]. The biocompatibility and strength of the genipin-crosslinked gelatin hydrogels have been demonstrated, they remained stable even after three months of incubation under cell culture conditions. To achieve conductive properties, poly(3,4-ethylenedioxythiophene) polystyrene sulfonate (PEDOT: PSS) modified with a gelatin-based solution was used as the electrode material [49].

Fig. 2a illustrated the fabrication of conductive hydrogel pillar electrodes. The fabrication process required a few steps employing a PMMA mold, simplified from our earlier study [24]. After adding PDMS and PMMA molds, an aqueous mixture of conductive hydrogel solution was dropped into the holes and incubated for 2 ​h for cross-linking. After one day of drying at room temperature, two conductive hydrogel pillar electrodes were obtained by peeling the entire glass substrate from the PMMA mold (Fig. 2b and c). SEM photos of hydrogel pillar electrodes in section and side view demonstrate their morphologies, which feature a porous internal structure and rough surface topography (Fig. 2d, e and Fig. S1). The hydrogel also exhibited stable rheological characteristics as determined by the examination of the hydrogels' storage (G′) and loss modulus (G″) at various angular frequencies. As shown in Fig. S2a, the G′ of different concentrations of hydrogel was greater than the G″, indicating that all of the hydrogels can form a stable colloid. In addition, excellent elastic recovery was seen in the results of the creep recovery process, the unrecoverable creep was very low, notably in the gelatin group and the 0.1 ​wt % PEDOT: PSS group (Fig. S2b).

Using AFM, the mechanical characteristics of wet hydrogel scaffolds were identified. The findings demonstrate that hydrogels' Young's modulus drops when increasing the concentration of PEDOT: PSS (within 0.1–0.3 ​wt %), ranging from 27.7 ​± ​1.7 ​kPa to 59.0 ​± ​5.7 ​kPa (Fig. 2f and Fig. S2c). Additionally, we used the CCK-8 assay to measure the viability of cells at various PEDOT: PSS doses. At concentrations no greater than 0.3 ​wt %, there was no discernible cytotoxicity (Fig. S2e). These results confirm that the conductive hydrogel pillar electrodes were soft and biocompatible in cultivation conditions. However, the hydrogels with 0.2–0.3 ​wt % PEDOT: PSS concentration are too soft to make pillar electrodes, compared with the concentration of 0.1 ​wt %. Therefore, the conductive hydrogel with 0.1 ​wt % PEDOT: PSS was used in the following experiments when preparing pillar electrodes. Importantly, the Young's Modulus of the hydrogel (0.1 ​wt %) did not change significantly for up to 1 month in culture (Fig. 2g).

The conductivity measurements were further performed with varied concentrations of PEDOT: PSS in hydrogels. As shown in Fig. S2d, the corresponding increase of conductivity can be achieved when increasing PEDOT: PSS from 0.1 to 0.3 ​wt %. However, the improvement is not very drastic, there was no significant difference among groups 0.1 to 0.3 ​wt %. By using electrochemical impedance spectroscopy (EIS), the impedance of gelatin-based conductive hydrogels was indirectly assessed. According to Fig. 2h and Fig. S3b, the conductive hydrogels' total impedance value (considered a measure of material resistance) was lower than that of the pure gelatin-based group. This finding was in line with many earlier studies and suggested that PEDOT: PSS could improve the electrical conductivity of the gelatin-based hydrogels [50]. CV tests confirmed that the electrical conductivities of the hydrogels were consistent with the results of the EIS (Fig. 2i). In addition, similar to Young's modulus, the conductive properties did not change significantly for up to 1 month (Figs. S3c and d).

### 3.3 Tissue generation in the heart-on-chip

**Fig. 3:**
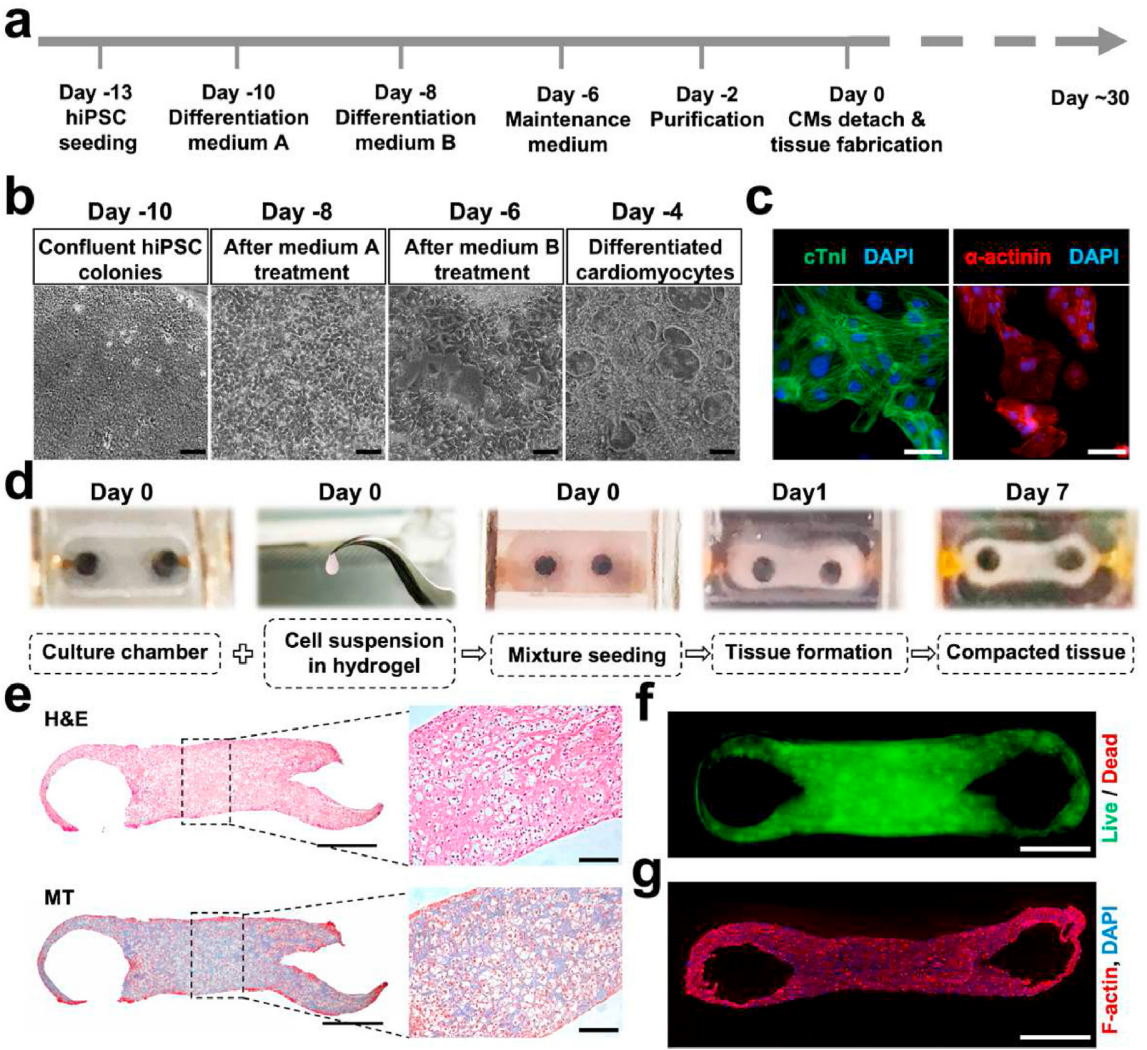
Cardiac tissue can be fabricated around hydrogel pillars. (a) Cardiomyocyte differentiation protocol outline. (b) Bright-field photos of cell morphology during cardiomyocyte differentiation. Scale bar: 100 ​μm. (c) cTnI, α-actinin and DAPI staining of differentiated cardiomyocytes. Scale bar: 20 ​μm. (d) Process for generating aligned cardiac tissues. (e) Representative images of newly formed cardiac tissue stained with hematoxylin-eosin (H&E) and Masson's trichrome (MT). Scale bar: 500 ​μm (100 ​μm for magnified images). (f) Representative live/dead image of formed cardiac tissue. Scale bar: 500 ​μm. (g) F-actin and DAPI staining. Scale bar: 500 ​μm.

In terms of maturity and functionality, significant advantages have been found in 3D cardiac cultures over 2D [51,52]. Furthermore, the incorporation of several heart cell types in 3D cultures replicates the *in vivo* microenvironment and encourages the systematic arrangement of cardiac organoids in culture [53]. To build a genuine co-culture system in the chip, we combined cardiac tissues that were made using cFBs and hiPSC-CMs together with collagen hydrogel. Using a PSC Cardiomyocyte Differentiation Kit, cardiomyocyte differentiation was carried out in accordance with the manufacturer's instructions (Fig. 3a and b). Cells that spontaneously contracted were seen starting on day 8 after differentiation stimulation (Fig. 3c). To ensure uniform cardiac tissue fabrication, we optimized the process by determining the CMs and cFBs ratio at 9:1 and collagen concentration at 3 ​mg/mL. After cell seeding, a compacted macroscopic cardiac tissue in the chip could be observed on day 1 (Fig. 3d and Figs. S4a and b). For the purpose of examining the structure of the cardiomyocytes, H&E and MT staining were performed. As shown in Fig. 3e, results showed that cells were evenly distributed throughout the cardiac tissue, reflecting the homogeneity of the tissue and no clusters formation. A live/dead staining assay conducted 1 day after the generation of cardiac tissue showed good biocompatibility (Fig. 3f). Uniform longitudinal alignment was also observed after 1 week in culture (Fig. 3g).

### 3.4 The electrical stimulation prompted the cardiac maturation

**Fig. 4:**
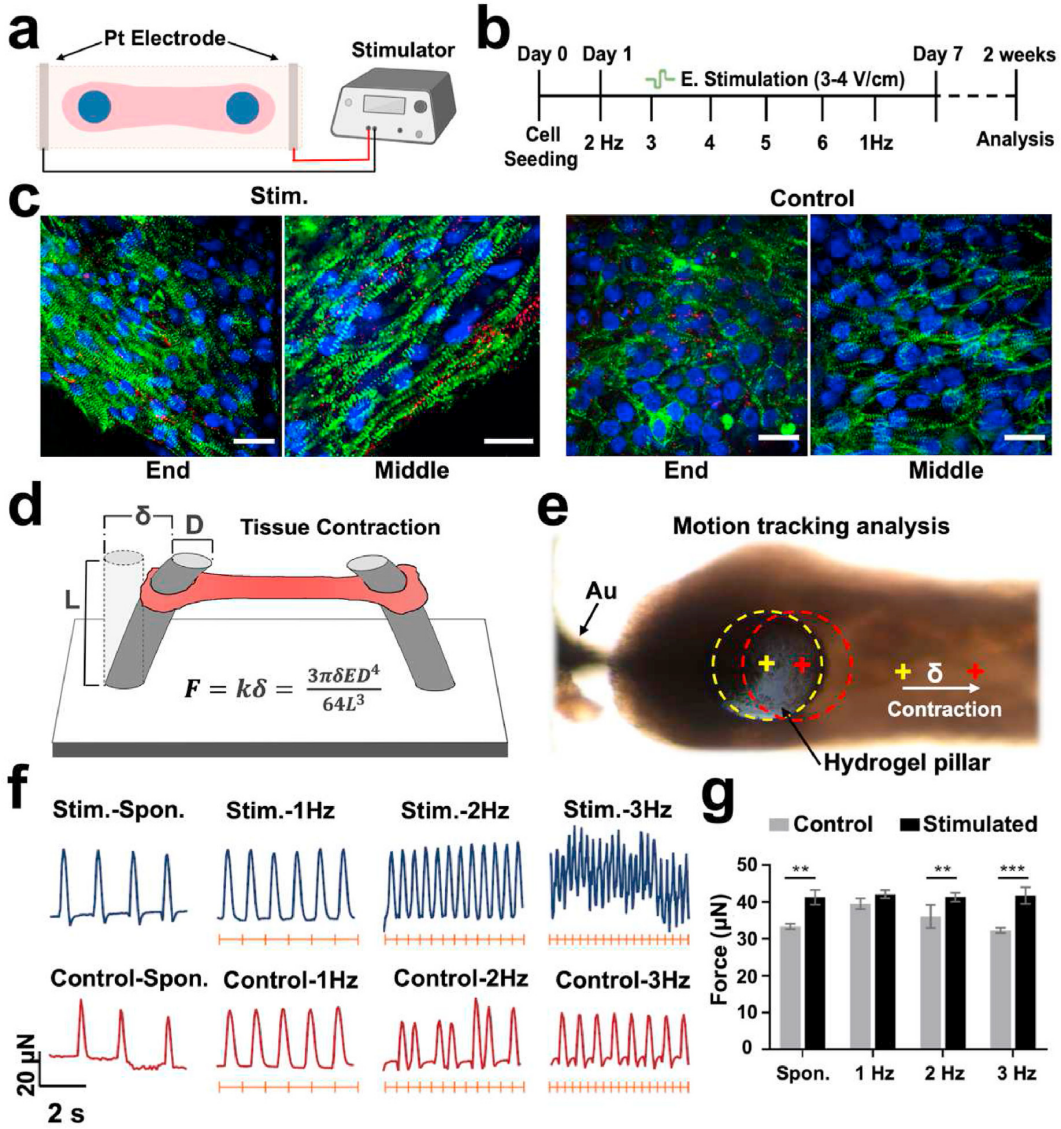
Impact of electrical stimulation on cardiomyocyte alignment and contractile behavior. (a) Electrical pacing diagram for cardiac tissue. (b) Experimental setup for applying electrical stimulation to the tissue. (c) Typical immunostaining images of unstimulated and stimulated tissues counterstained with DAPI and stained for sarcomeric α-actinin (green) and connexin-43 (red). Scale bar: 20 ​μm. (d) The contractile force is calculated by measuring the lateral deflection of each hydrogel pillar (δ) and multiplying it by the pillar's spring constant (k). (e) Motion tracking analysis of cardiac tissue. (f) Contraction force of control and stimulated tissues paced with 1, 2, and 3 ​Hz, calculated using software for tracking the displacement of hydrogel pillars. Pacing frequency is shown by the orange tick marks. (g) Peak contraction force statistics of control and stimulated tissues paced with 1, 2, and 3 ​Hz (n ​= ​10/11 for normal and stimulated tissues, respectively). All data are represented as means ​± ​SD.

Typically, the tissues start beating on day 1 and undergo a 7-day compaction process after seeding (Fig. S4c). During this process, a short-term gradual upscaling electrical stimulation loading was performed to warm up the *in situ* electrical pacing function of the heart-on-chip (Fig. 4a), which is beneficial to the maturation of cardiomyocytes [7]. On the first day, the chip device was linked to a cardiac stimulator to electrically pace the heart tissue at a frequency of 2 ​Hz, followed by 1 ​Hz step-up stimulation every day until 6 ​Hz. After that, a 1 ​Hz stimulation was maintained to prevent abnormal effects such as arrhythmogenicity that may occur after high-frequency stimulation (Fig. 4b) [24]. After one week of culture, as a result of the various pacing circumstances, the tissues displayed notable variations in the cytoskeletal architecture. Fixing the tissue and staining it for α-actinin revealed that all tissues had much more sarcomere formation than on day 1 of the experiment. But it also showed clear variations in sarcomeric alignment between the stimulated and control groups, in which stimulated cardiac tissue established apparent anisotropy of sarcomeres and exhibited more connexin-43 expression and directional alignment (Fig. 4c, Fig. S5). This suggested that the heart-on-chip device integrated with electrical stimulation could stimulate immature cardiomyocytes to develop into a more mature phenotype.

In terms of contractility, the contractile activity of the tissues cultivated under conditions with or without electrical stimulation was evaluated and analyzed using custom-developed software for monitoring the movement of the hydrogel pillars. The tissue's contractile force, which was computed using the hydrogel pillars' stiffness k, is proportional to the displacement of the tip of the pillars (Fig. 4d and e). The contractile force generated by the cardiac tissues is similar to many other reports, ranging from 10 to 100 ​μN [25,54]. The contraction force was greater in the stimulated group versus the unstimulated group under both spontaneous and paced conditions. Furthermore, the stimulated cardiac tissues could generate a synchronous beating pattern responding to the applied frequencies of 3 ​Hz, whereas controls were less able to respond at a frequency of 2 ​Hz (Fig. 4f). This high-frequency stimulus responsiveness also indicated the mature characteristics [24].

It's interesting to note that up to 1.5–1.8 ​Hz, all unstimulated tissues could be paced; beyond this frequency, tissues could no longer match the external pacing rate. Every other pulse was skipped, resulting in a beating frequency that was half of the applied frequency (Fig. 4f). A graph of peak contraction force for each condition shows the relative magnitude of the difference between the control and stimulated groups (Fig. 4g). The normalized contraction force observed in stimulated tissues was significantly improved (Fig. S6). However, despite signs of increased maturation, such as better sarcomere development and higher contraction force, no positive force-frequency association was seen in the stimulated tissues. Other researchers have also reported similar results, and one possible explanation was a lack of mature calcium handling performance [55]. The cardiac tissues in the present study may benefit from electrical stimulation for more extended periods or treatment with maturation-promoting compounds like dexamethasone, triiodothyronine, and insulin-like growth factor 1 [56,57].

### 3.5 Noninvasive monitoring of calcium handling

**Fig. 5:**
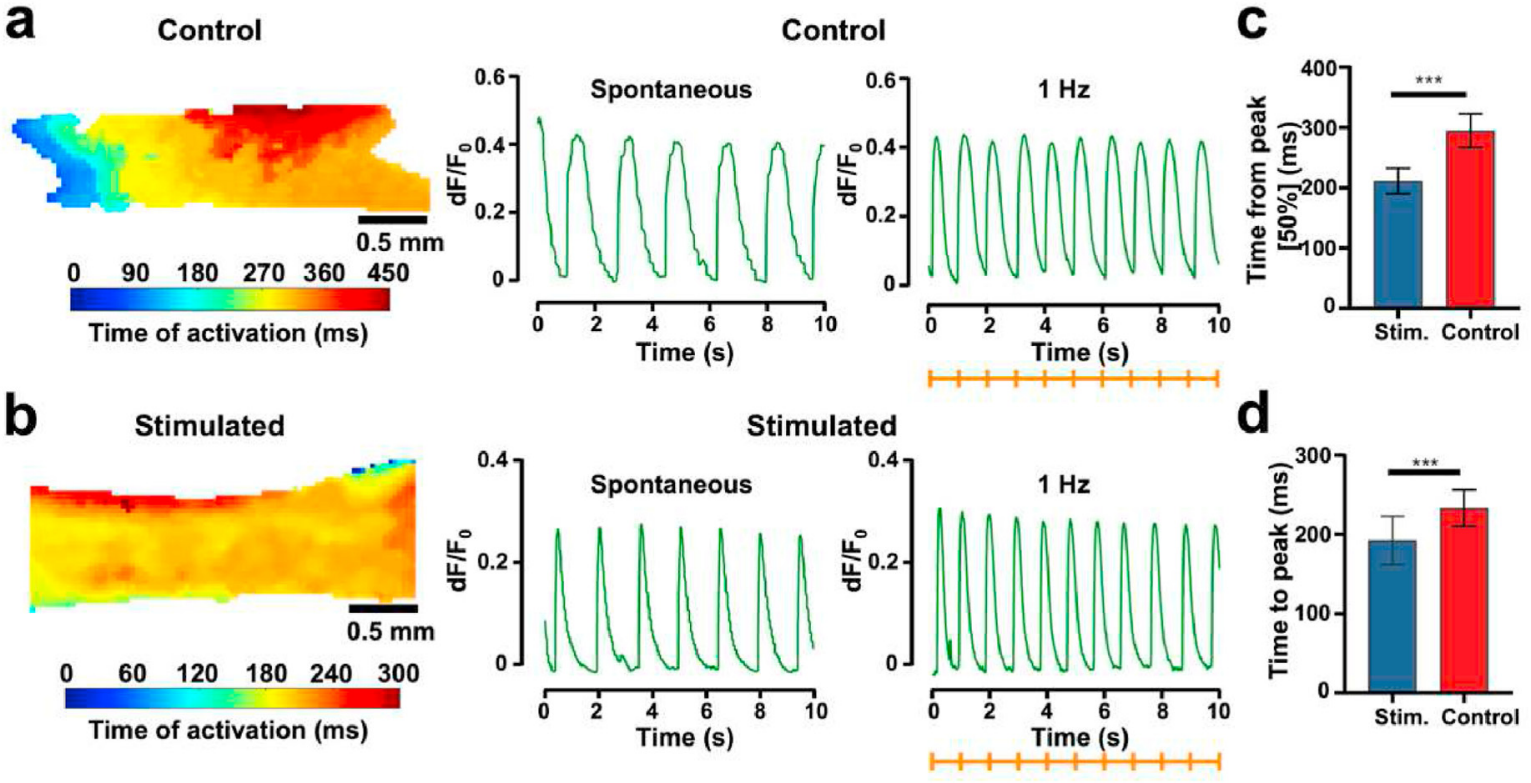
Electrical stimulation prompted the maturation of calcium handling in the heart-on-chip platform. (a–b) At 1 ​Hz field stimulation, calcium wave propagation and typical calcium transient curves from the tissue were obtained. The color map demonstrated the calcium wave's temporal delay as it moved through the tissue. The orange tick marked indicates pacing frequency. (c–d) Assessment of calcium transport rate at 1 ​Hz. Time to peak: the process of releasing calcium from the sarcoplasmic reticulum; Time from peak [50%]: calcium ion reuptake from the sarcoplasmic reticulum (n ​= ​12/15 for control and stimulated tissues, respectively). All data were represented as means ​± ​SD.

We next assessed the calcium handling after the course of electrical stimulation. Uniaxial conduction was visible using calcium imaging across the heart tissues, and the stimulated tissues showed improved synchronization of calcium waves compared to the controls (Fig. 5a and b). Specifically, the stimulated tissues presented a significant shortening of the wave width, revealing a more rapid calcium handling capacity. On the contrary, some abbreviated irregular calcium peaks could be observed in the control groups (data not shown). Moreover, the unstimulated tissues showed prolonged calcium release and decay rates, reflecting immature sarcoplasmic reticular function (Fig. 5c and d). In summary, these data demonstrated the ability of our heart-on-chip platform to noninvasively record cardiac tissue contractility and calcium signals.

### 3.6 Recording of cardiac tissues electrophysiology and drug responses

A key advantage of our heart-on-chip platform is the ability to record the electrophysiologic characteristics of cardiac tissues in real-time. As a proof of concept, we exploited the two conductive hydrogel pillars to monitor tissue's action potential propagation activity as an extracellular field potential and detected the changes in electrophysiology when administered with drugs that cause well-known electrical alterations.

**Fig. 6:**
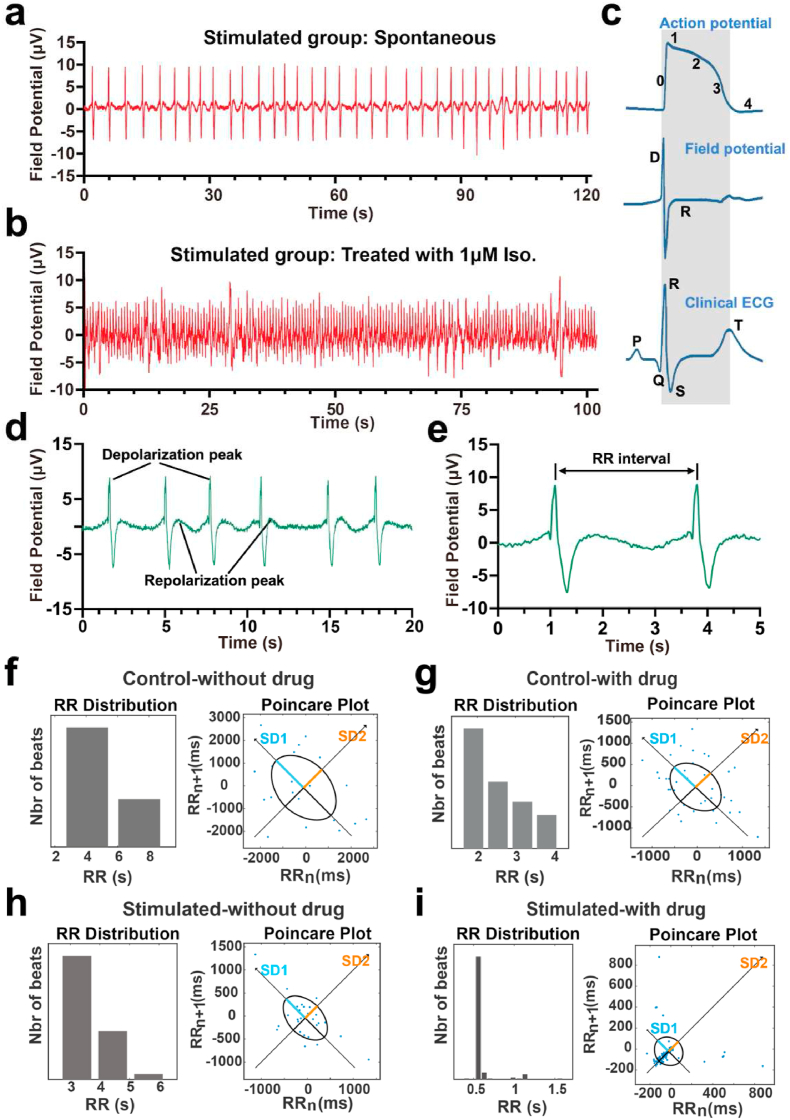
Monitoring cardiac tissues' electrical activity in the heart-on-chip device under dynamic conditions. (a–b) Tissue extracellular field potentials were captured using hydrogel electrodes in stimulated groups. Electrical activity under spontaneous and 1 ​μM isoprenaline conditions was measured. (c) Action potential propagation activity was detected as an extracellular field potential, which is very similar to clinical ECG. (d) The electrical activity of the tissues showed spontaneous, synchronized beating, as indicated by the depolarization and repolarization peaks in the recording curves. (e) 5-s zoomed-in plot of d. (f–i) Poincaré plot results were obtained by calculating the corresponding RR intervals. After isoprenaline treatment, the representative stimulated cardiac tissues had a more robust and regular spontaneous beating behavior and reached a higher and sustained synchronized activity. SD1: Poincaré plot short term variability; SD2: Poincaré plot long term variability.

As shown in Fig. 6, in accordance with the contraction behavior evidenced by optical observation, rhythmic peaks were seen from the control and stimulated tissues with amplitudes ranging from −10 μV to 10 ​μV. This amplitude is smaller than some published reports, probably due to the increased electrode impedance of hydrogel compared to standard metal electrodes [13,35]. Nonetheless, the local field potential was clearly evident with minimal noise because of the performance of the conductive hydrogel pillar electrodes, which enabled reliable signal capture (Fig. 6a). Similar to a clinical ECG, we could also recognize a typical cardiac field potential profile that consists of a depolarization spike and repolarization phases (Fig. 6c–d). As expected, the stimulated tissues displayed a more robust and consistent spontaneous beating tendency, further confirming the guiding effect of electrical stimulation on tissue beating behavior (Fig. 6a and Fig. S7a). To test the sensitivity of signal acquisition and the local drug administration's capacity in the heart-on-chip device, we stimulated the tissues with isoprenaline. Isoprenaline is a synthetic β-adrenergic receptor agonist, which is recognized to have a positive chronotropic impact, increasing the heartbeat's frequency [58]. 1 ​μM isoprenaline was dynamically administered through the channel that permitted fluidic connection with the cardiac tissues. An escalating pattern of beating frequency was observed in both stimulated and control tissues (Fig. 6b and Fig. S7b). However, the stimulated tissues demonstrated a more physiological drug response. The corresponding captured signals showed a typical positive chronotropic response, as evidenced by the regular frequency (∼2 ​Hz) of the spikes (Fig. 6b). The stimulated tissues also displayed a gradual slowing and cessation of beating behavior after receiving additional verapamil (Fig. S8). The time between waves (inter-beat (RR) interval) can be translated to the rate of the heart for any pair of beats (Fig. 6e) [59]. In order to measure the RR interval to track the cardiac tissue on the environment and physiological stimulus, these results were further confirmed by Poincaré plots (Fig. 6 f-i), pointing out shortened RR intervals and lower RR intervals variability after dosing in the stimulated tissues. Altogether, we showed that synthetic cardiac tissues respond positively to isoprenaline in terms of chronotropy, demonstrating the recapitulation of physiological characteristics. It is worth noting that in the drug administration test, we employed an injection pump for dynamic perfusion culture, which can constantly update the medium and alleviate the problem of drug concentration decline in the medium caused by drug absorption by PDMS. Our findings confirmed the applicability of our platform for signal detection and drug testing. Moreover, the detection function of arrhythmia events was also available due to the continuous monitoring capability of our heart-on-chip platform.

## 4 Conclusion

In the present study, we reported a novel heart-on-chip platform that generates 3D cardiac tissue and was capable of applying electrical stimulation to improve functional performance. A major innovation of this study was the demonstration that soft conductive hydrogel pillar electrodes were promising 3D structures for both relevant electrophysiology measurement and reproducible contractile quantification. Moreover, we further illuminated that the stimulated tissues respond to isoprenaline with an increasing beat frequency trend, demonstrating the dependability of this heart-on-a-chip platform for use in cardiac toxicity screening and medication efficacy testing. Overall, the designed system can be regarded as a flexible, mass-produced platform for pharmaceutical development and disease modeling.

## Credit author statement

Feng Zhang: Conceptualization, Methodology, Investigation, Data curation, Formal analysis, Visualization, Funding acquisition, Writing – original draft, Writing – review & editing; Hongyi Cheng: Methodology, Investigation, Data curation, Writing – review & editing; Kaiyun Qu: Methodology, Investigation, Data curation; Xuetian Qian: Methodology, Investigation; Yongping Lin: Investigation, Data curation; Yike Zhang: Investigation; Sichong Qian: Investigation; Ningping Huang: Project administration, Supervision; Writing – review & editing; Chang Cui: Project administration, Supervision, Funding acquisition, Writing – review & editing; Minglong Chen: Project administration, Funding acquisition, Supervision, Writing-review & editing.

## Declaration of competing interest

The authors declare that they have no known competing financial interests or personal relationships that could have appeared to influence the work reported in this paper.

## Data Availability

Data will be made available on request.

## Appendix A Supplementary data

The following files are available free of charge: SEM characterization of hydrogel scaffolds; Characterization of hydrogel scaffolds; Electroconductive properties of the conductive hydrogels; Generation of human cardiac tissues; Polar histograms display primary orientations extracted from high-magnification (20 ​× ​) images of anisotropic cardiac macrofilaments; Force on Day 7 normalized by cross-sectional area; Monitoring cardiac tissues’ electrical activity in control groups; Electrical activity of stimulated cardiac tissue under 1 ​mM verapamil administration condition.
