# Supplementary material for: Continuous contractile force and electrical signal recordings of 3D cardiac tissue utilizing conductive hydrogel pillars on a chip
Source: Mater Today Bio. 2023 Apr 6;20:100626. doi: 10.1016/j.mtbio.2023.100626 (PMC10130626; doi:10.1016/j.mtbio.2023.100626)
Supplement: Multimedia component 1 [file mmc1.docx]

**Supporting Information**

**Continuous contractile force and electrical signal recordings of 3D cardiac tissue utilizing conductive hydrogel pillars on a chip**

*Feng Zhang ^1,2, †^,* *Hongyi Cheng ^1,3, †^, Kaiyun Qu ^2, †^, Xuetian Qian ^4^, Yongping Lin ^1^, Yike Zhang ^1^, Sichong Qian ^5^, Ningping Huang ^2,*^, Chang Cui ^1,*^, Minglong Chen ^1,2,3,6,*^*

^1^Department of Cardiology, The First Affiliated Hospital of Nanjing Medical University, Nanjing, Jiangsu 210000, China

^2^State Key Laboratory of Bioelectronics, School of Biological Science and Medical Engineering, Southeast University, Nanjing 210096, China

^3^Gusu School, Nanjing Medical University, The Affiliated Suzhou Hospital of Nanjing Medical University, Suzhou, Jiangsu 215002, China

^4^Department of Gastroenterology, Nanjing First Hospital, Nanjing Medical University, No. 68 Changle Road, Nanjing 210006, China

^5^Department of cardiac surgery, Beijing Anzhen Hospital, Beijing, 100029, China

^6^Key Laboratory of Targeted Intervention of Cardiovascular Disease, Collaborative Innovation Center for Cardiovascular Disease Translational Medicine, Nanjing Medical University, Nanjing, Jiangsu 210000, China

*Corresponding author

E-mail addresses: chenminglong@njmu.edu.cn (M. Chen); cuichang@njmu.edu.cn (C. Cui); nphuang@seu.edu.cn (N. Huang).

^†^ These authors contributed equally to this work.

This file contains: Supplementary Figure S1-S8.


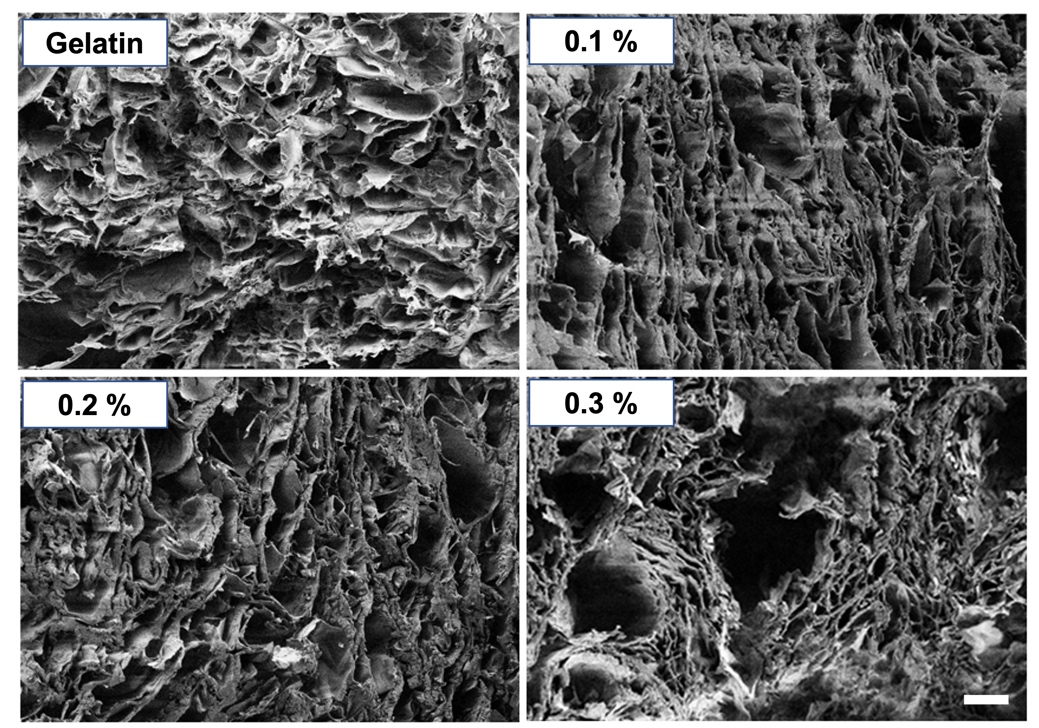


Figure S1. SEM characterization of hydrogel scaffolds. SEM cross-sectional images of gelatin hydrogels with different concentration of PEDOT:PSS.
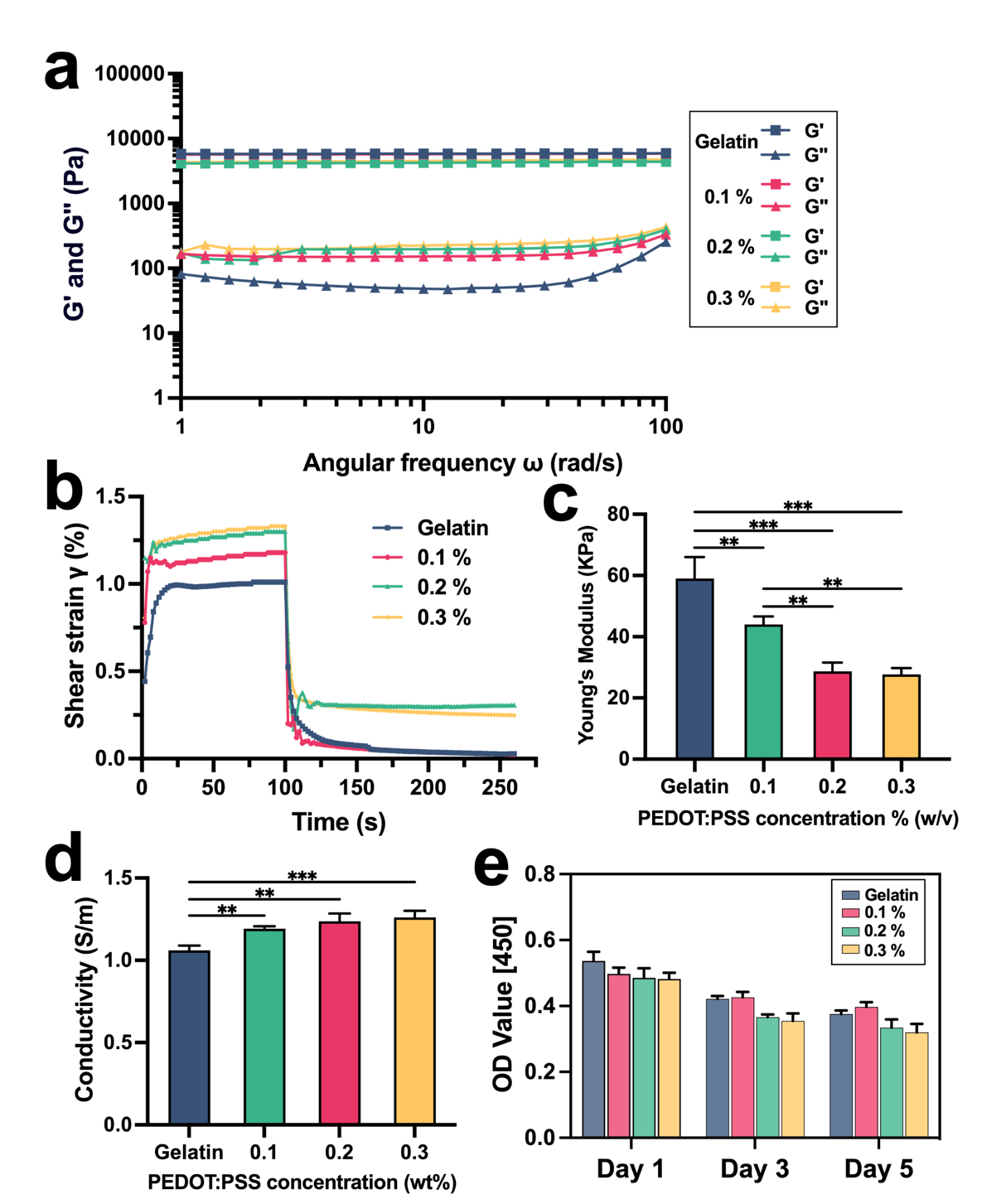


Figure S2. Characterization of hydrogel scaffolds. (a) Amplitude sweep performed with the angular frequency increasing from 1 to 100 rad/s. (b) Creep recovery curves of the hydrogels with different concentration of PEDOT:PSS. (c)Young’s modulus of gelatin-based hydrogels incorporated with PEDOT: PSS after cross-linking measured by AFM. (d) The conductivity of the hydrogels with different concentration of PEDOT:PSS. (e) Viability of cardiac cells on the hydrogel scaffolds with different concentration of PEDOT:PSS.


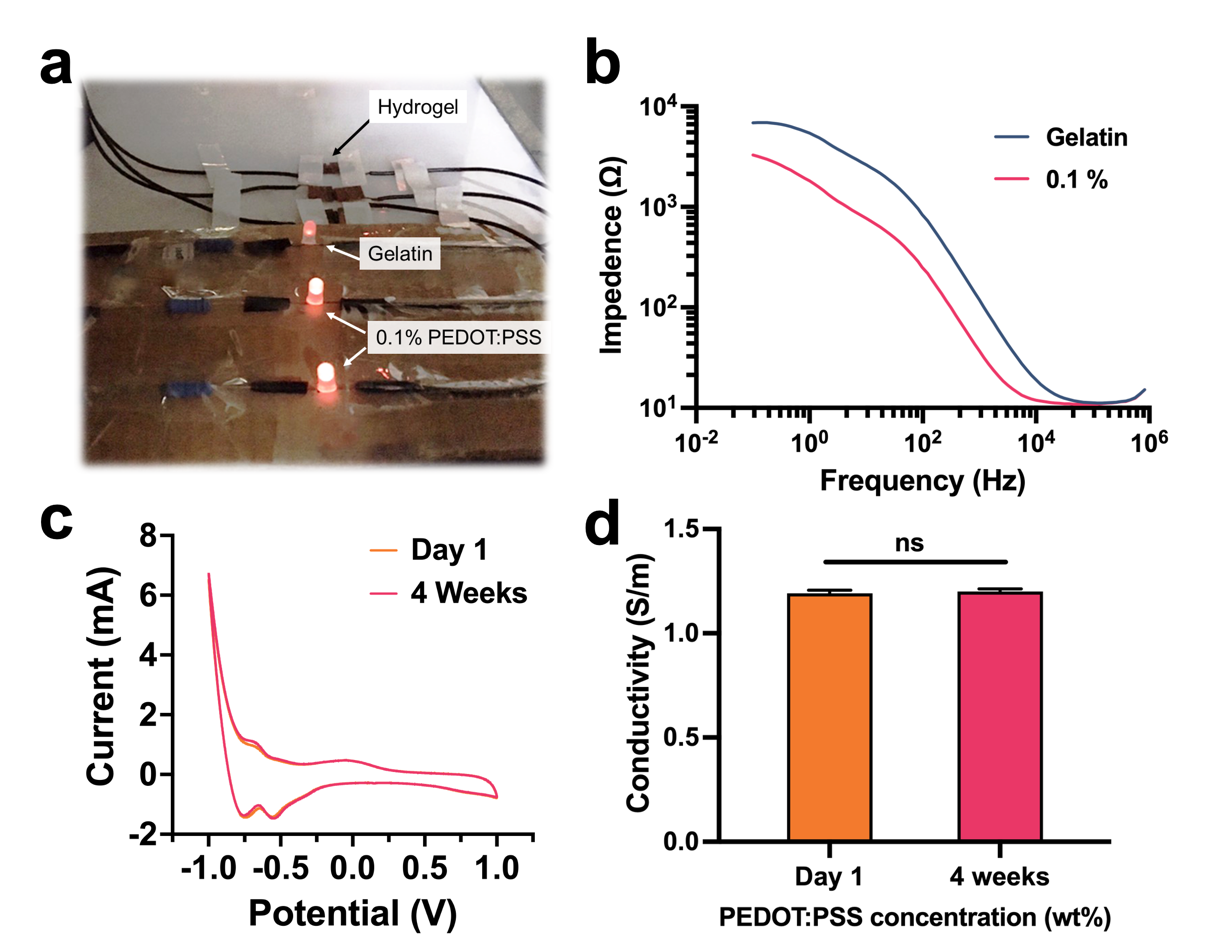


Figure S3. Electroconductive properties of the conductive hydrogels. (a) Photo demonstrating the good conductivity of the hydrogel with 0.1 wt% PEDOT:PSS. (b) Electrical impedance of the conductive hydrogels. (c) CV curves of the 0.1 wt% PEDOT:PSS content hydrogel before and after 4 weeks. (d) Conductivity characterization of the 0.1 wt% PEDOT:PSS content hydrogel before and after 4 weeks.


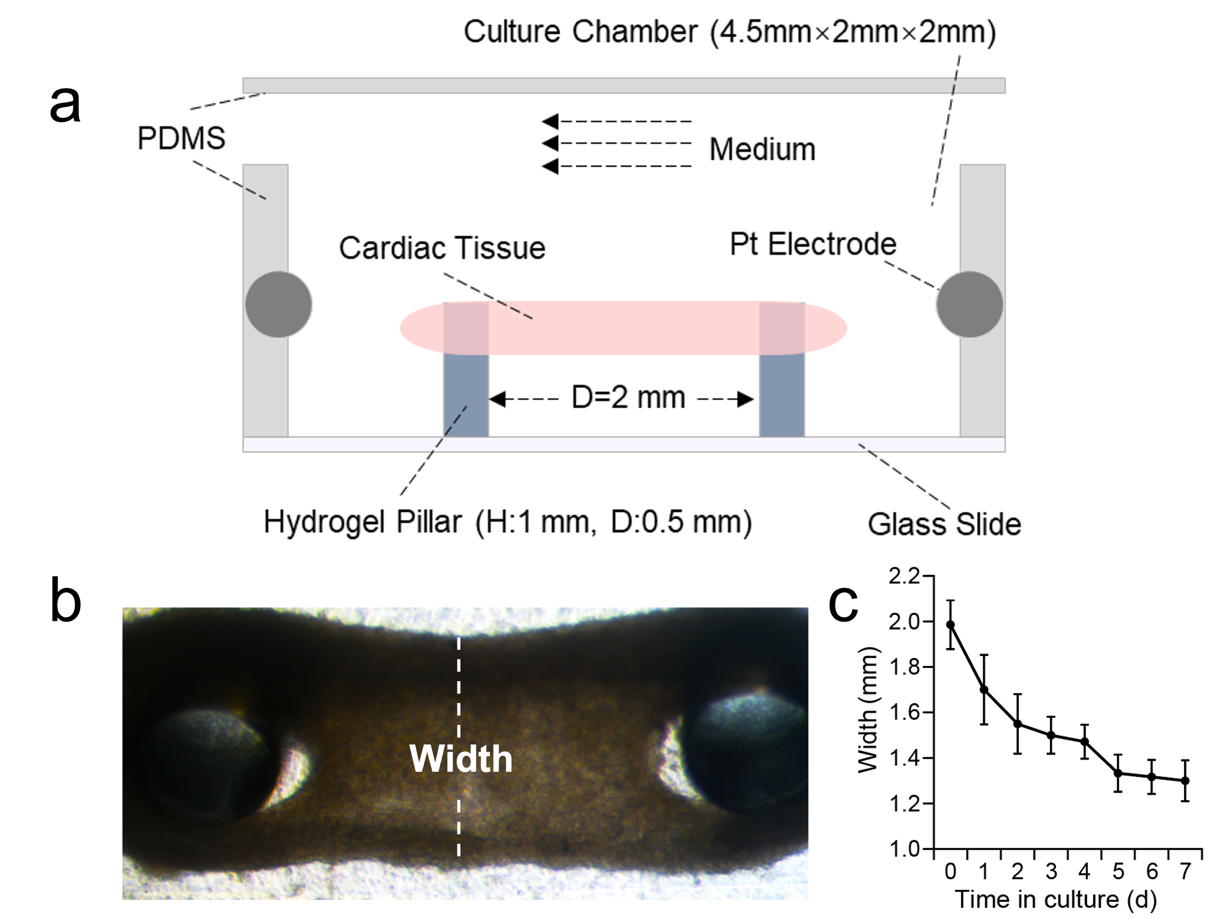


Figure S4. Generation of human cardiac tissues. (a) Schematic. Representative tissue in the heart-on-chip platform. (b) Brightfield image of cardiac tissue. (c) Quantification of gel compaction on the indicated days of culture. (n = 6 (day 0-4), n = 5 (days 5-7)).


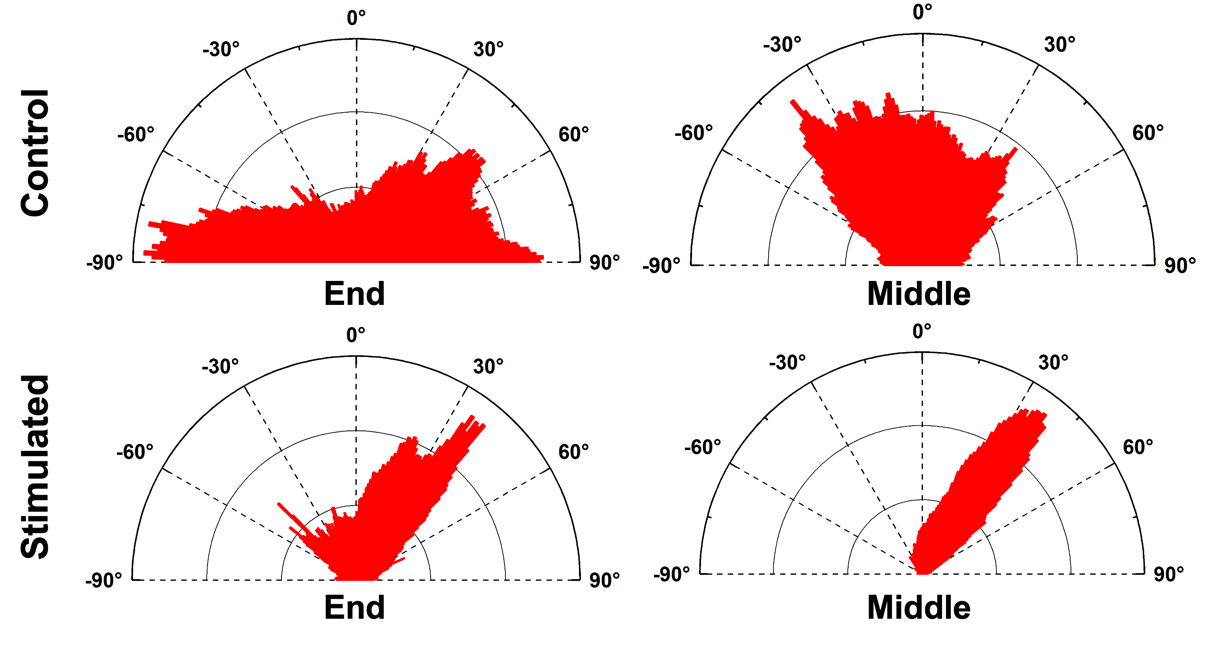


Figure S5. Polar histograms display primary orientations extracted from high-magnification (20×) images of anisotropic cardiac macrofilaments.


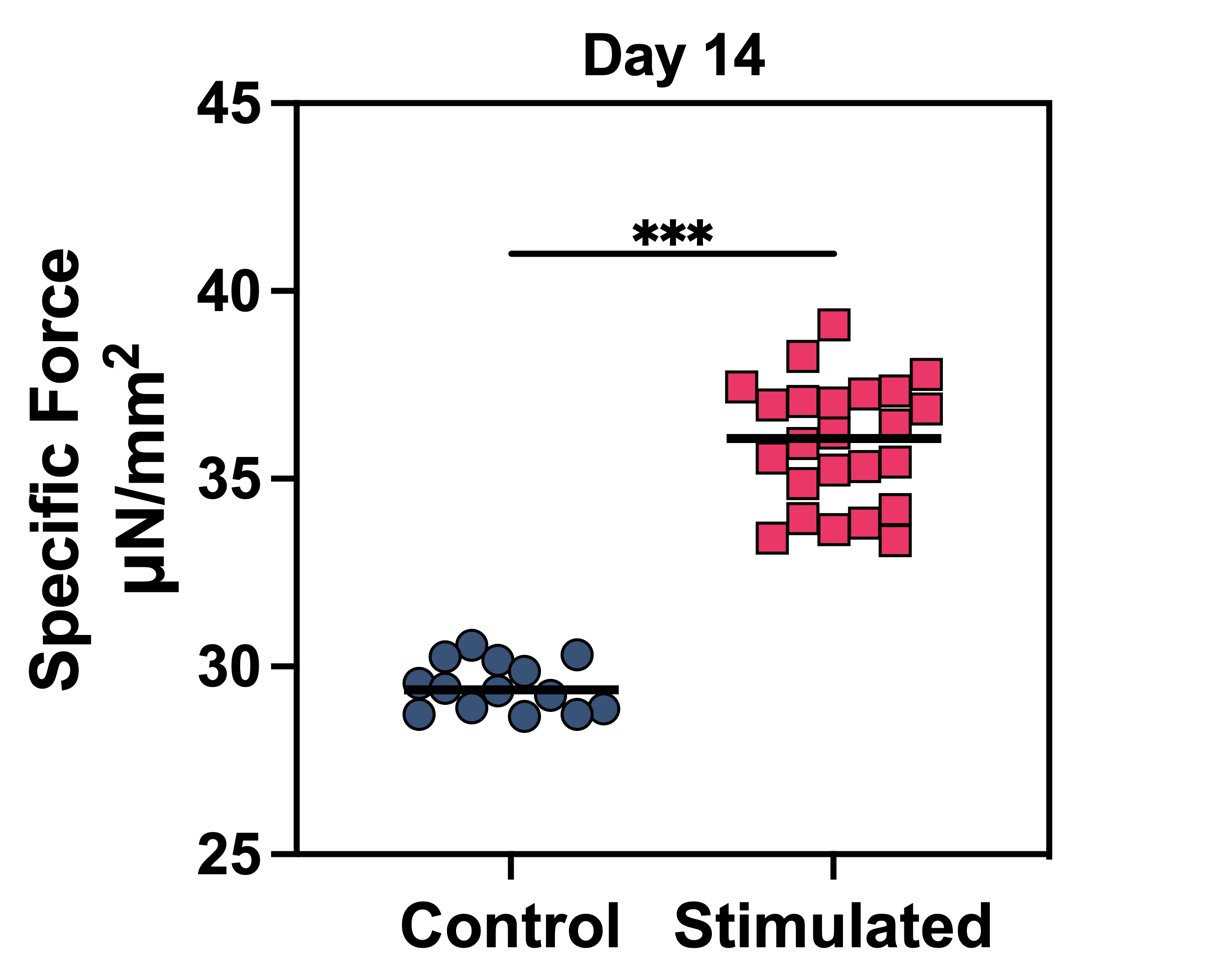


Figure S6. Force on Day 7 normalized by cross-sectional area.


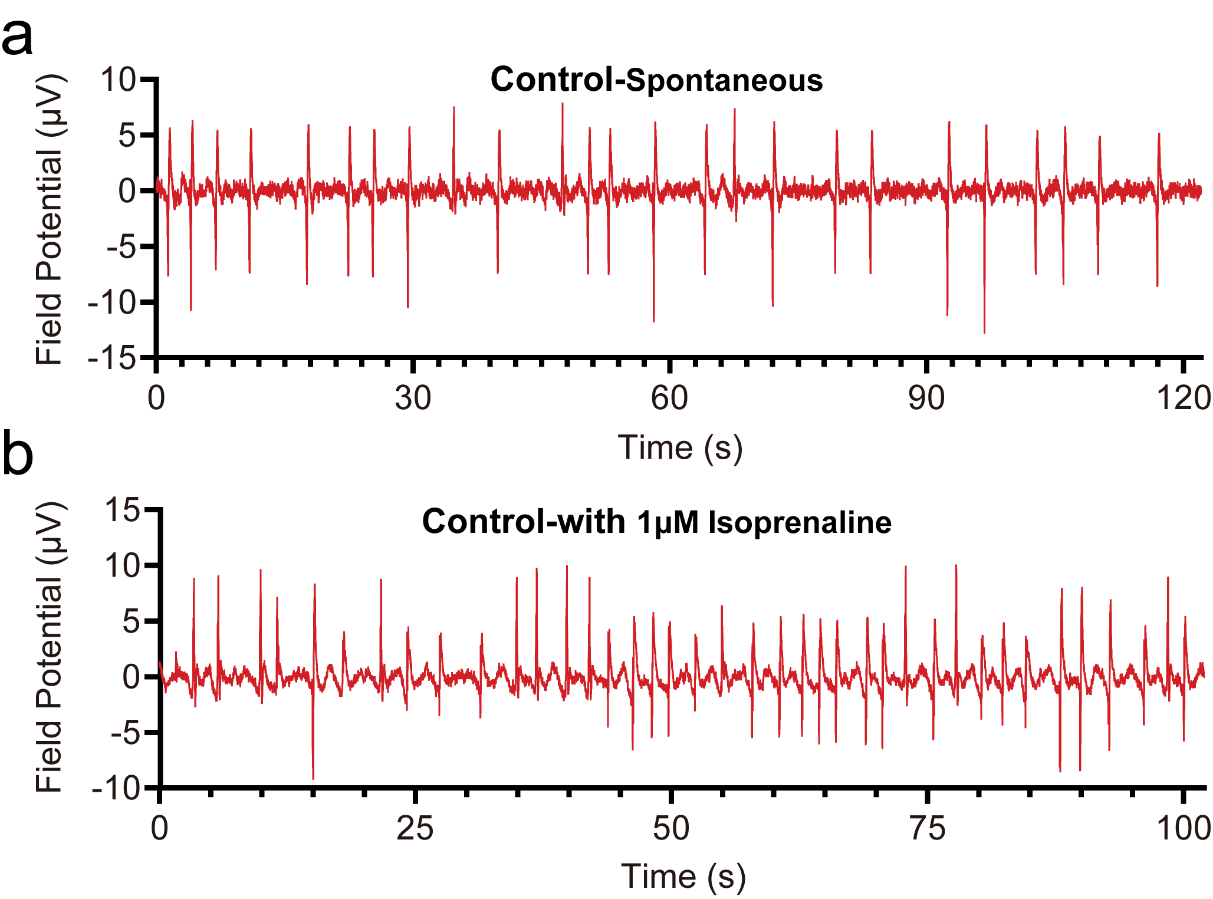


Figure S7. Monitoring cardiac tissues’ electrical activity in control groups. (a-b) Electrical activity under spontaneous and 1 μM isoprenaline condition was measured.


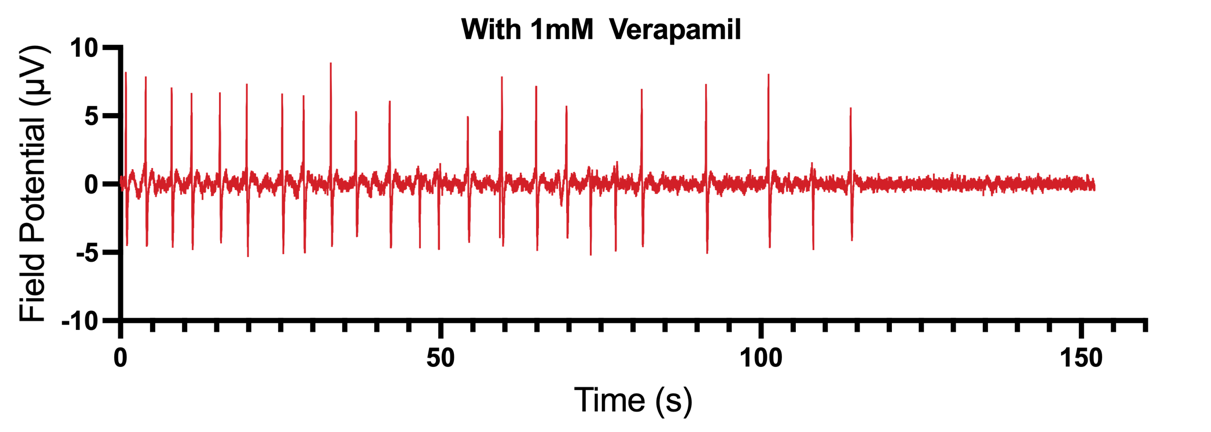


Figure S8. Electrical activity of stimulated cardiac tissue under 1 mM verapamil administration condition.
